# Nitrogen loss from karst area in China in recent 50 years: An in‐situ simulated rainfall experiment's assessment

**DOI:** 10.1002/ece3.3502

**Published:** 2017-10-24

**Authors:** Xianwei Song, Yang Gao, Sophie M. Green, Jennifer A. J. Dungait, Tao Peng, Timothy A. Quine, Bailian Xiong, Xuefa Wen, Nianpeng He

**Affiliations:** ^1^ Key Laboratory of Ecosystem Network Observation and Modeling Institute of Geographic Sciences and Natural Resources Research CAS Beijing China; ^2^ College of Resources and Environment University of Chinese Academy of Sciences Beijing China; ^3^ University of Exeter Exeter UK; ^4^ Department of Sustainable Soils and Grassland Systems Rothamsted Research Okehampton UK; ^5^ State Key Laboratory of Geochemistry Geochemistry Institute of CAS Guiyang, Guizhou China; ^6^ College of Resources and Environment Zunyi Normal College Zunyi Guizhou China

**Keywords:** karst, nitrogen, simulated rainfall, subsurface runoff, surface runoff

## Abstract

Karst topography covers more than 1/3 of the People's Republic of China in area. The porous, fissured, and soluble nature of the underlying karst bedrock (primarily dolomite and limestone) leads to the formation of underground drainage systems. Karst conduit networks dominate this system, and rainfall takes a crucial role on water cycle at China karst area. Nitrogen loss from the karst system is of particular concern, with regard to nutrient use efficiency as well as water quality, as much of the karst system, including steeply sloping terrain, is used for intensive agriculture. We use simulated rainfall experiments to determine the relationship between rainfall and nitrogen loss at typical karst slope land and then estimate nitrogen loss from the karst soil. The results show that both surface runoff and subsurface runoff have a significant linear correlation with rainfall at all studied sites. Subsurface runoff is larger than surface runoff at two karst sites, while the opposite is true at the non‐karst site. Exponential function satisfactorily described the correlation between rainfall and nitrogen concentrations in runoff. Nitrates accounted for 60%–95% of the dissolved nitrogen loss (DN, an index of N‐loss in this research). The estimated annual N‐loss load varies between 1.05 and 1.67 Tg N/year in the whole karst regions of China from 1961 to 2014. Approximately, 90% of the N‐loss load occurred during the wet season, and 90% of that passed through the subsurface. Understanding the processes and estimating N‐loss is highly valuable in determining long‐term soil security and sustainability in karst regions.

## 1 INTRODUCTION

Karst topography is formed by the dissolution of soluble rock, usually carbonate rocks such as dolomite, limestone, and gypsum, and carbonate rocks outcrop across ~11% of the world's ice‐free land area (Badman, 2010; Dürr, Meybeck, & Dürr, 2005). It covers extensive parts of China (~3,440,000 km^2^), especially in southwestern China where rapid and intensive land use change has caused severe system degradation within only the last 50 years. Karst rocky desertification is characterized as the processes of transformation of vegetation and soil covered karst landscape into exposed basement rocks, and has become the most serious pressure in southwestern karst area in China (Gao et al., 2013a; Gao et al., 2013b; Wang et al., 2004b). The ongoing desertification has been suggested to have significant implications through changes in evaporation that could affect the timing and location of monsoon rains in Southwest China, such as the net radiation, evaporation, and rainfall were reduced (Gao et al., 2013a; Gao, Yu, & He, 2013b).

Karst topographies are characterized by eroded surface features and extensive subsurface drainage or karst conduit network (KCN). KCN is defined as a complex system constituted of soil porosity, underground fissures, cenotes, and funnels. KCN is mainly influenced by rock weathering and soil erosion, and chemical dissolution during hydrological processes, especially rainstorm in karst areas, contribute much to it (Chang et al., 2015; Song et al., 2017). Water and nutrients could enter the KCN through soil and are transported to the subterranean system easily, but the rates of transport and response times to precipitation are poorly understood in South China karst zone, and the consequences for the geochemical cycles are profound and difficult to predictable. Rainfall simulations are often used to investigate both surface and subsurface runoffs, hydraulic conductivity, soil erosion, and nutrient loss under controlled conditions (e.g., Fu et al., 2015, 2016; Gao et al., 2009, 2010; Taucer et al., 2008). Using this approach, Taucer et al. (2008) found much water bypassing the litter and soil layers via macropore pathways while a portion of water remain as matrix and conduit storage. Similarly, Teixeira and Misra (2005) reported that N‐loss accompanied by sediment loss was richer in eroded soils than those of the uneroded soils. They also found a ratio between N‐loss and sediment loss is 0.16% (0.0016 kg N‐loss with 1 kg sediment loss) (Teixeira and Misra, 2005). Nutrient loss through surface runoff has been studied relatively extensively compared to subsurface runoff, and few studies have focused on the epikarst. Yue et al. (2015) demonstrated that nitrate in the HouZhai catchment was mainly impacted by manure sources during the dry season and influenced by a mix of chemical fertilizer and manure during the wet season. Li et al. (2013) assessed that water and nutrient loss were seriously hindering natural vegetation growth in southwestern China. Both ecosystem research for macroscopic scale and cellular functions study for microcosmic scale found N limitation at karst area in China (Kang et al., 2015; Zhang et al., 2015). These findings highlight that nutrients leached from the epikarst system have different loss pathways and influences on karst ecosystem.

Soil formation in karst areas is slow due to its base geology, and soil loss by accelerated soil erosion by water from the steeply sloping terrain is widespread. The geochemical consequences of erosion vary and are based on the characteristics of the (1) epikarst, (2) the surface of the karst, and (3) the geological structure of the drainage basin and the underlying bedrock (Wang et al., 2004; Zhang et al., 2014). Anthropogenic factors (e.g., agricultural cultivation, grazing, deforestation, and burning) also significantly influence erosion and drainage in karst systems (Gao et al., 2017; Wang et al., 2016). Aridity and water shortages are intensified by the thin and spatially variable soils and fast percolation through soil and rock fissures in China karst zone (Wang et al., 2004b). During heavy rainfall events (i.e., during monsoonal wet season), there is significant runoff and an increased potential for soil nutrient loss. This has profound implications for soil and water quantity and quality and ecosystem service provision, including water provision and food production.

Overuse of mineral fertilizers in China has been the subject of recent scrutiny because of the negative impacts on human and environmental health, and the increasing disconnect between application rates and yield benefits: from 1996 to 2005, grain yields in China increased by only 3.5%, while use of the chemical fertilizers supplying N, increased by 24% (Zhang et al., 2014). Environmental pollution related to poor nutrient management has become a serious issue over last 20 years, with N concentrations in surface and ground water regularly exceeding WHO recommendations (Zhu and Chen, 2002). Thus, the Chinese Government's current 5‐year plan has set a stringent target “Zero Increase Action Plan” for fertilizers by 2020. Leaching of soluble N species (mainly nitrate; NO_3_^−^) from the thin, calcareous soils of karst systems may reduce the ability of the soil to provide vital ecosystem services, for example, nutrients and water regulation and supply, carbon sequestration, and food production (Zhang et al., 2011a, 2011b; Huebsch et al., 2014; Liang et al., 2005; Zhu et al., 2016). Therefore, a fuller understanding of the controls on erosional processes and the correlation between water and N cycle in karst topographies is imperative in terms of long‐term soil and water security in these fragile regions.

**Figure 1:**
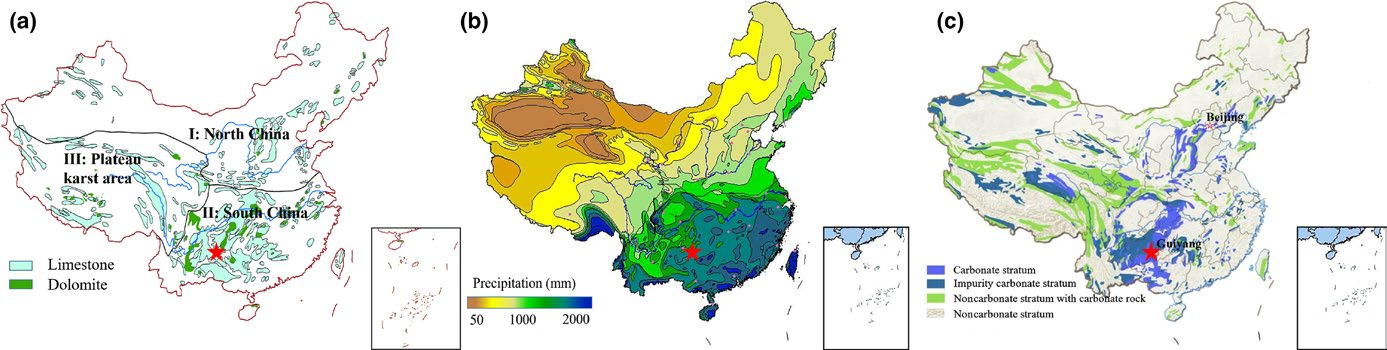
(a) Karst geomorphology regions of China; (b) Average rainfall distribution in recent years; (c) Carbonate rocks distribution in China. Study sites location marked with red star

In this paper, we tested the hypothesis that N losses from karst landscapes differ between parent geologies and calculated the magnitude of N loss in China karst area. There are three simplified karst zones based on bedrock type and precipitation, with the concentrated area of karst located in the South China (Figure 1a and Table 1). We performed in situ rainfall simulations at different bedrock types in the karst region of South China, and pay close attention to the upper soil and interface of soil‐bedrock, which dominate the main KCNs. Different intensities of simulated rainfall (30, 45, 70, 90, and 120 mm/hr) performed during a specified time of an hour to investigate surface and subsurface hydrological processes. We studied key parameters of rainfall‐runoff conversion process and the amount of N‐species lost quantitatively. Contrastive discussion between karst sites and non‐karst sites also presented. For further synthesis of above factors, we estimated the annual and monthly variations in dissolved nitrogen (DN) loss (N‐loss) from karst regions in China between 1961 and 2014 according to monthly mean precipitation data.

**Table 1:** Distribution proportion of dolomite and limestone in three karst regions in China

Karst regions	Dolomite distribution proportion	Limestone distribution proportion	Total area (10^4^ km^2^)
North China	0.1	0.9	102.7
South China	0.2	0.8	127.5
Plateau area	0.1	0.9	114.5

## 2 MATERIALS AND METHODS

### 2.1 Study areas

Three experimental sites, established in Shawan (SW), Chenqi (CQ) and Huangtupo (HTP), are located within Puding County, Guizhou, China (26°15′N, 105°43′E) (Figure 3a). The lithology of SW and CQ is dominated by dolomite and limestone (both from the Guanling formation of the middle Triassic), respectively. The site at HTP is covered with mixed soils mainly include sedimentary red soil and yellow soil (red–yellow soil). SW and CQ served as two different typical karst zones and both the soil thickness is about 0.6 m. Meanwhile, HTP served as the control site and the soil thickness is over 1.5 m. HTP is bare cropland while both SW and CQ are uncultivated land covered with grass. Table 2 shows detailed soil properties (Table 2). As an in‐situ experiment, there is no chemical fertilizer and manure applied within a year. The nutrient sources mainly are atmospheric deposition for the region, biological N fixation, and N release from bedrock weathering.

**Table 2:** Soil characteristics at experiment sites. d(0.5) means median particle diameter (mm)

Sample site	SW			CQ			HTP		
Lithology	Dolomite			Limestone			Mixed red and yellow soil		
Slope degree	20°			12°			15°		
Sample depth (cm)	0–10	10–20	30–50	0–10	10–20	30–50	0–10	10–20	30–50
pH	7.82	7.85	7.90	7.52	7.70	7.72	7.86	7.84	7.82
Soil bulk density	1.08	1.75	1.76	1.27	1.23	1.33	1.08	1.27	1.16
TC %	2.89	6.74	11.25	3.53	8.14	2.92	2.56	2.82	1.75
TN %	0.25	0.51	0.09	0.30	0.55	0.25	0.21	0.17	0.16
NO_3_^−^‐N (mg/kg)	1.41	2.16	0.82	0.57	0.43	0.55	1.54	0.91	0.67
NH_4_^+^‐N (mg/kg)	4.24	2.67	1.72	2.29	1.33	0.91	1.50	1.24	0.87
Particle size analysis %									
d (0.5)	5.68	7.96	15.32	6.72	5.69	5.40	6.39	7.62	10.05
<0.002 mm	13.94	10.31	6.79	13.67	14.75	16.36	17.67	14.22	11.39
0.002~0.02 mm	78.74	75.95	54.86	72.23	76.28	75.31	71.81	74.72	69.24
>0.02 mm	7.32	13.74	38.36	14.10	8.97	8.33	10.52	11.05	19.37

### 2.2 Experimental plot design

**Figure 2:**
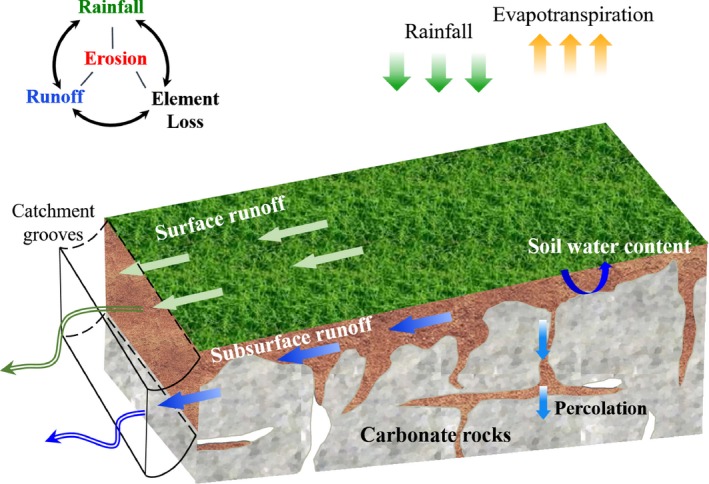
Schematic diagram of karst structure and runoff paths

**Figure 3:**
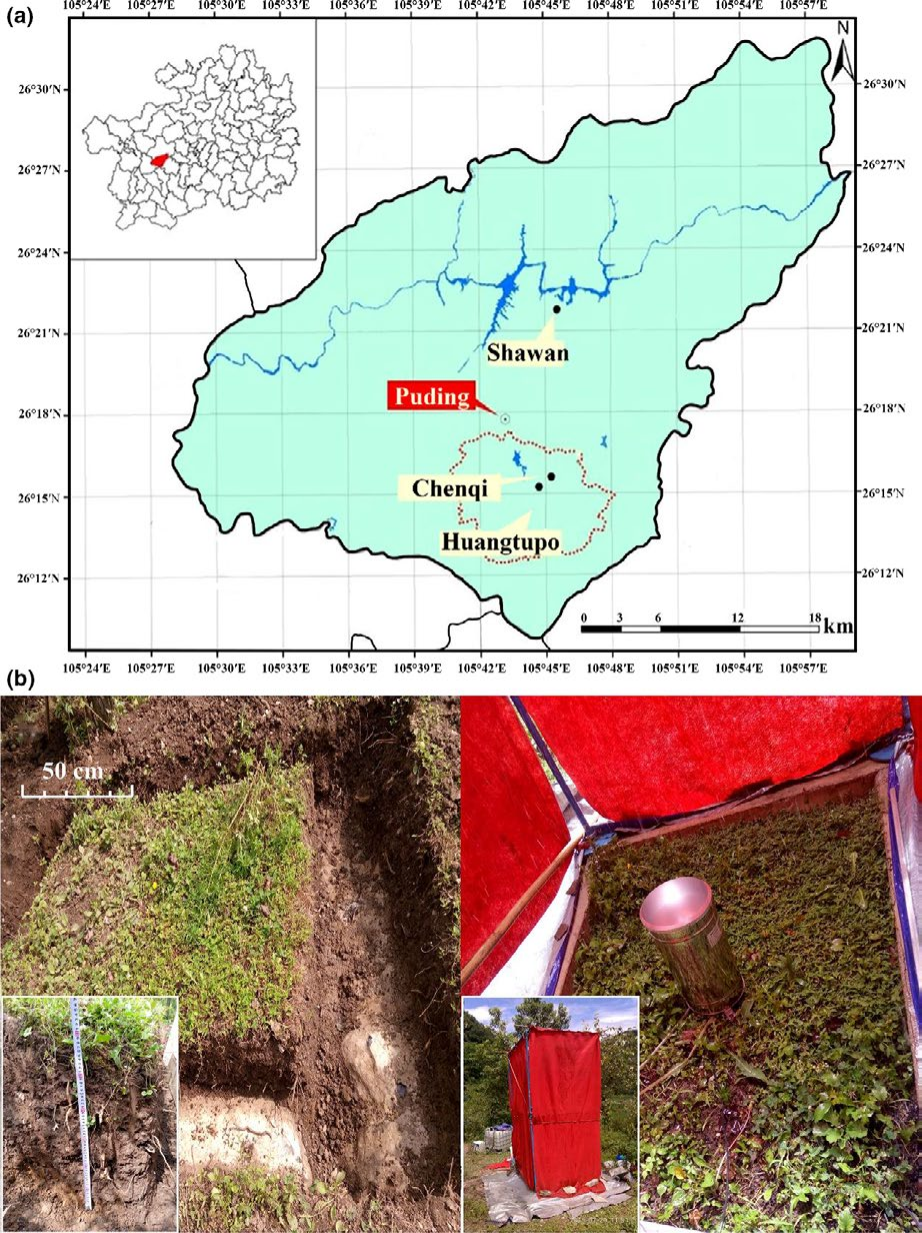
(a) Study sites location in Puding County, Guizhou; (b) Size of experimental plot (left) and Simulated rainfall system (right)

The experimental plots were established on slopes of 12–20° in SW, CQ, and HTP in June 2016 (during wet season), which is representative of common karst slope areas used for agriculture (Chen et al., 2012). Each study sites has three contiguous replicated rectangular plots, and all the nine plots have same size (1.66 × 1.16 × 0.6 m (length × width × depth), active area is 1.89 m^2^) (Figure 3b). Catchment grooves were constructed at the downhill end of each plot and connected, by 4 m long closed pipes, to two plastic buckets, one that collected surface runoff at 10 cm depth and one that collected subsurface runoff at 60 cm depth (Figure 2).

### 2.3 Simulated rainfall system

Customized rainfall simulators (NLJY‐10, Nanjing Nanlin Electronic Co., Ltd, Nanjing, China) were used to generate to simulated rainfall of varying intensities with a uniformity greater than 86% measured by the manufacturer. The simulator was 2.0 × 1.5 × 3.0 m (length × width × height), and surrounded with wind barriers (sheeting—see Figure 3b). The sizes of three nozzles are 1, 2, and 3 mm, respectively. A rain gauge was used to measure and record rainfall intensity. Each simulated rainfall lasted for a consistent time of 60 min, and five different intensities were applied during the experiment (30, 45, 70, 90, and 120 mm/hr). Then, we use this rainfall gradient to establish quantitive correlation between rainfall depth and runoff, rainfall depth and N‐loss concentration as well. A minimum rest time of 24‐hr between each simulated rainfall was designed to ensure that there were minimal carry over affects between experimental runs. A HOBO U30 micro automatic meteorological station (Onset, MA, USA) was used to monitor whether the soil return to its initial, pre‐experimental moisture content. Two sensors were buried at the center of each plot at depths of 10 and 20 cm, respectively. A waterproof tarpaulin was used to eliminate natural rain interference during the rest time.

### 2.4 Soil and water analysis

Soil was sampled at three layers (0–10, 10–20, 30–50 cm). We used a foil sampler (100 ml) for soil sampling and calculated soil bulk density by weighting before and after oven drying at 105°. Slope degree was measured by leveling instrument and protractor. C and N content were analyzed by Vario MAX CN (Elementar, Germany). Nitrate (NO_3_^−^) and ammonium (NH_4_^+^) content were analyzed by Segmented Continuous Flow Analyzer (Futura, Alliance, France) after extraction by K_2_SO_4_ (0.5 mol/L). Particle size analyzed by Mastersizer 2000 laser particle analyzer (Malvern, UK).

Water samples (100 ml) of both surface runoff and subsurface runoff were collected at intervals between 1 and 3 min (*n *=* *10). After each rainfall event, the total runoff volume was noted. pH was determined at time of sample collection (Ultrameter II^™^, Myron L Company, USA). All samples were stored at 4°C after being filtered through a 0.45 μm Mixed Cellulose Ester under vacuum. DN, NO_3_^‐^‐N, and NH_4_^+^‐N in each sample were measured using Segmented Continuous Flow Analyzer (Futura, Alliance, France). Performance of all instrumental methods was checked using synthetic standard reference materials. The time between initiation of simulated rainfall and appearance of the first runoff is noted as runoff yielding time (*T*_y_). All the time is recorded during each rainfall event.

### 2.5 N‐loss estimated method

As rain gauges measure rainfall in mm, we converted water volume (ml) to runoff depth (mm), which was defined as the depth of the water runoff through 1 m^2^ during a temporal period. Equations (1), (2), and (3) were used to calculate runoff coefficients:

(1) $$Q=\frac{W}{S}$$

(2) $$P=\int_{0}^{t}I_{t}dt$$

(3) $$R_{c}=\frac{Q}{P}$$

where *Q* is the runoff depth (mm), *W* is the total runoff volume (L) in a definite area, *S* is the effective rainfall area (m^2^); *P* is the simulated rainfall (mm); *I*_t_ is the real‐time rainfall intensity recorded by hyetometrograph (mm/hr); *t* is the rainfall duration (hr); *R*_c_ is the runoff coefficient.

Thereafter, the data were used to train models (Linear Fit, Quadratic Polynomial Fit, and Exponential Decay 1 Fit) to express the correlation between rainfall and runoff, and rainfall and DN concentration.

(4) $$Q=f\left(x\right)=\{\begin{array}{cc} kx+t & x\geq P_{c}\\ Ax^{2}+kx+t & x\geq P_{c}\\ 0 & x\leq P_{c} \end{array}$$

(5) $$C=f\left(x\right)=\{\begin{array}{c} kx+t\\ Ax^{2}+kx+t\;x\\ Aexp\left(-\frac{x}{k}\right)+t \end{array}$$

In Equation (4) and (5) *A*,* k,* and *t* are all fitting coefficients according to different models, *x* represents rainfall depth (mm), *P*_c_ is the hypothetical threshold rainfall that will trigger runoff (mm), *C* is the concentration of DN.

Daily precipitation data (National Meteorological Information Center of the China Meteorological Administration, http://cdc.nmic.cn/home.do accessed) were used to calculate the mean precipitation for the karst region (interpolation, and Inverse Distance Weighting approaches were used). The rainfall could be distinguished as erosive rainfall and nonerosive rainfall based on whether soil will loss due to the rainfall erosion, and the estimated rainfall amount standard of erosive rainfall was about 10–15 mm in China (Xie et al., 2000, 2016). However, this research just focus on DN coupling with runoff, which means that N‐loss could occur even if the rainfall is much lower than erosive rainfall standard. Therefore, the erosive rainfall standard is invalid in this research, and we propose using minimum rainfall (*P*_min_) of runoff yielding to distinguish whether N‐loss happen. We get rid of the daily precipitation which is lower than *P*_min_, and then calculate the month rainfall depth (*P*_month_) through summation of the remaining daily precipitation data (*P*_day_). The relevant expressions as follow:

(6) $$P_{\min}=MIN\left\{I_{t}T_{y}\right\}$$

(7) $$P_{\mathrm{month}}=SUM\left\{\begin{array}{c} P_{\mathrm{day}}\;\;\;\left(P_{\mathrm{day}}>P_{\min}\right)\\ 0\;\;\;\;\;\;(P_{\mathrm{day}}<P_{\min}) \end{array}\right\}$$

where *P*_min_ means the minimum rainfall depth (mm) while surface or subsurface runoff start yielding; *T*_y_ is the lag time between runoff yielding and rainfall starting. Monthly N‐loss flux (mg/m^2^) estimated by Equation (7). We substitute the *P*_month_ into fitting Equation (4) and (5) to calculated runoff and corresponding DN concentration. Then, monthly N‐loss flux can be calculated by following Equation (8):

(8) $$L_{i}=Q_{\mathrm{SFi}}C_{\mathrm{SFi}}+Q_{\mathrm{SSi}}C_{\mathrm{SSi}}$$

where *L*_i_ is the monthly N‐loss flux (mg/m^2^); *Q*_SF_ is runoff depth (mm)for surface runoff per month; *Q*_SS_ is runoff depth (mm) for subsurface runoff; *C*_SF_ is DN concentration ([DN], mg/L) in surface runoff; *C*_SS_ is [DN] (mg/L) in subsurface runoff.

(9) $$L=aS\sum_{i=1}^{12}L_{\mathrm{ai}}+bS\sum_{i=1}^{12}L_{\mathrm{bi}}$$

where *a* is the proportion of the karst region underlain by dolomite; *b* is the proportion of the karst region underlain by limestone; *S* is the karst area in China (km^3^); *L* is the potential annual DN loss (kg/year).

All statistical analysis was completed using Microsoft Excel 2016, SPSS 21.0, and Origin 9.0. Linear model, quadratic model, and exponential decay model were performed for regression analysis between rainfall and runoff, as well as rainfall and N‐loss. Pearson test was used to describe the correlation level of the fitting analysis results; One‐way ANOVA was used to analysis of variance.

## 3 RESULTS

### 3.1 Basic soil properties

The mean pH of three layers at each site shows SW > HTP > CQ at 0.05 level, even that the pH vary between a small range from 7.52 to 7.90. The soil bulk density of 10–20 cm layer and 30–50 cm layer at SW is much larger than other sites and layers, because SW has a thin soil layer contains lots of dolomite fragment at the middle and bottom of soil layer. SW and CQ have a significantly higher soil bulk density than HTP for the mean value of three layers. The total C (TC) and total N (TN) in different layers of soil are of great difference, which the layer 10–20 cm has maximum TC and TN at SW and CQ, and the layer 0–10 cm has maximum TC and TN at HTP.

### 3.2 Characteristics of runoff

#### 3.2.1 Runoff yielding time

**Figure 4:**
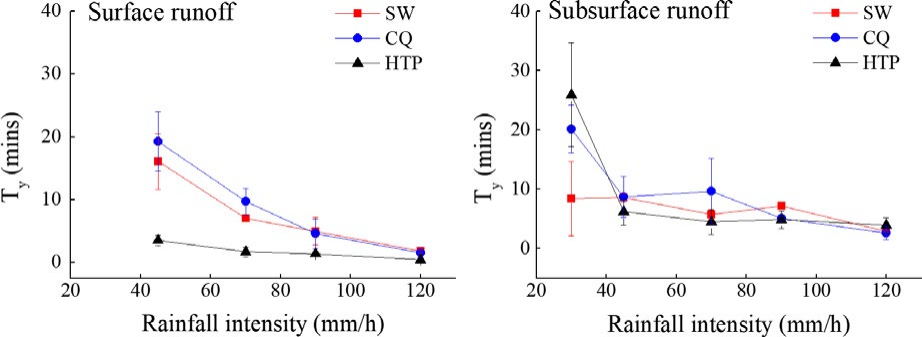
*T*_y_ of surface runoff and subsurface runoff under different rainfall intensities

Runoff yielding time decreased as simulated rainfall increased in intensity (Figure 4). This correlation is likely to be controlled by the physical properties of soil such as density, porosity, and permeability. SW, CQ, and HTP show no surface runoff yield when the simulated rainfall intensity is below 30 mm/hr. All surface *T*_*y*_ *s* for each rainfall intensity at HTP are under 5 min while T_y_s of SW and CQ decreasing gradually within 25 min with rainfall intensity increasing (Figure 4). When the simulated rainfall intensity was higher than 45 mm/hr, surface runoff occurs in all plots. We could infer that a threshold of rainfall intensity controls surface and subsurface runoff yielding events, which has a range of 30–45 mm/hr.

#### 3.2.2 Runoff discharge and coefficient

**Figure 5:**
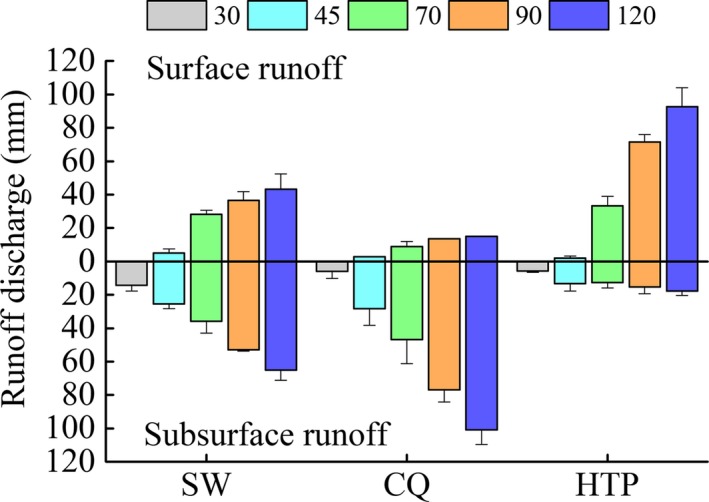
Total runoff depth of surface flow and subsurface flow. Different colors represent different rainfall intensities. Top and bottom parts represent surface runoff and subsurface runoff, respectively

**Figure 6:**
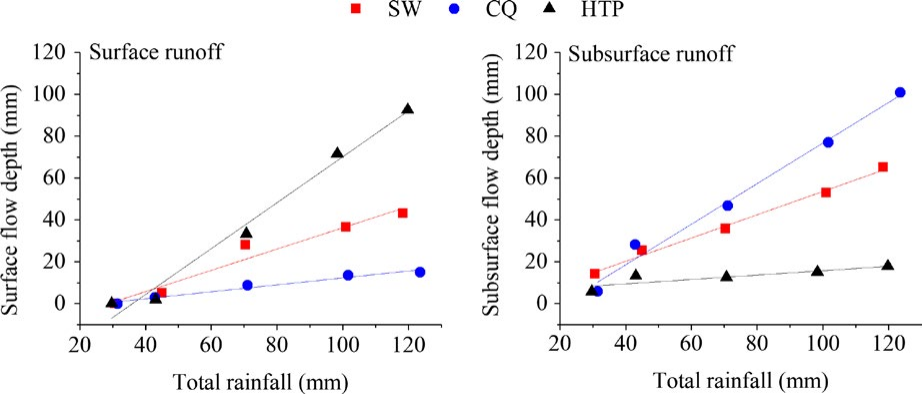
Linear fit of runoff with rainfall. For surface runoff, the fitting results are *y* = −14.58 + 0.51*x*,* y* = −4.23 + 0.17*x*, and *y* = −39.48 + 1.10*x* at SW, CQ, and HTP, respectively. For subsurface runoff, the fitting results are *y* = −1.99 + 0.56*x*,* y* = −20.19 + 0.97*x*, and *y *=* *5.29 + 0.10*x* at SW, CQ, and HTP, respectively

Volume and distribution of surface and subsurface runoff at three experimental sites show differences obviously, which displays increased runoff as simulated rainfall intensity increased (Figures 5 and 6). Surface runoff is highest at HTP and lowest at CQ (HTP > SW > CQ, *p *<* *.05) when rainfall intensity is over 70 mm/hr. Subsurface runoff, on the contrary, is highest at CQ and lowest at HTP (CQ > SW > HTP, *p *<* *.05). In other words, at SW and CQ, most of the runoff consisted of subsurface runoff, and in comparison at HTP, the majority of runoff consisted of surface runoff. As we expected, karst soil has high water permeability and water flows rapidly in the karst soil. As basement rock covered with only about 60 cm soil, water flows at the interface of rock and soil easily when infiltrating into this layer and then comes into being the main subsurface runoff.

When the simulated rainfall intensity is below 45 mm/hr, surface runoff is minimal and the runoff coefficients are low at all three sites (Table 3). Therefore, we infer that there is a minimum limit of rainfall intensity for surface runoff yielding. Dai et al. (2015) also found that there is no surface runoff when the rainfall intensity is small (30 and 50 mm/hr) using indoor simulated rainfall experiments. When the simulated rainfall is above 70 mm/hr, surface runoff coefficients are lower than subsurface runoff coefficients at SW and CQ; the inverse was true of HTP coefficients (*p *<* *.05; (Table 3). Peng et al. (2008) built six runoff plots at the same catchment with our CQ site and observed surface runoff coefficients of 0.01%–12.8% during July, 2007 to February, 2008 at a condition of natural rainfall. And it is also found that surface runoff coefficients increase rapidly when rainfall reach to 80–90 mm and change little when rainfall lower than 60 mm (Peng et al., 2008). Therefore, the mean surface runoff at CQ measured under simulated rainfall is close to that measured during actual rainfall events.

**Table 3:** Runoff coefficients of surface and subsurface runoff under different rainfall intensities (%)

Rainfall intensity (mm/hr)	Surface flow			Subsurface flow		
	SW	CQ	HTP	SW	CQ	HTP
30	0.0	0.0	0.0	46.0	18.4	19.3
45	11.3	6.7	4.5	56.4	65.9	30.6
70	40.0	12.4	47.1	50.8	65.8	17.7
90	36.2	13.3	72.7	52.3	75.6	15.3
120	36.5	12.1	77.3	55.0	81.6	14.8
Average	24.8	8.9	40.3	52.1	61.4	19.6

#### 3.2.3 Rainfall‐runoff correlation

Both linear and polynomial fits were explored to determine the correlation between total rainfall (mm) and runoff (mm). The linear fits of rainfall and surface runoff have good performance which *r* ^2^ is 0.94, 0.96, and 0.98 and corresponding Pearson correlation coefficients are 0.98, 0.99, and 0.99 for CQ, SW and HTP, respectively. Which the fit of rainfall and SS had an *r* ^2^ of 0.99, 0.98, and 0.68 and the corresponding Pearson correlation coefficients are 0.99, 0.99, and 0.87 at these sites, respectively. Although both linear and polynomial fits have fine fitting results, we prefer to use the linear fits (Figure 6) for the rainfall‐runoff process simulation because of its understandability and directness. As the absolute slope of the fitting equation represents the response degree of runoff to rainfall, Figure 6 shows that surface runoff changes more greatly at the non‐karst area (HTP). The subsurface runoff at the HTP was relatively low, and the fitting correlation with rainfall intensity is not pronounced as with the karst experimental sites (both *r* ^2^ and Pearson coefficient is lower than SW and CQ at 0.05 level). Then, we infer that subsurface runoff is the predominate pathway in karst areas.

### 3.3 Dissolved N‐loss along with runoff

#### 3.3.1 Dissolved N‐loss forms and concentration

**Figure 7:**
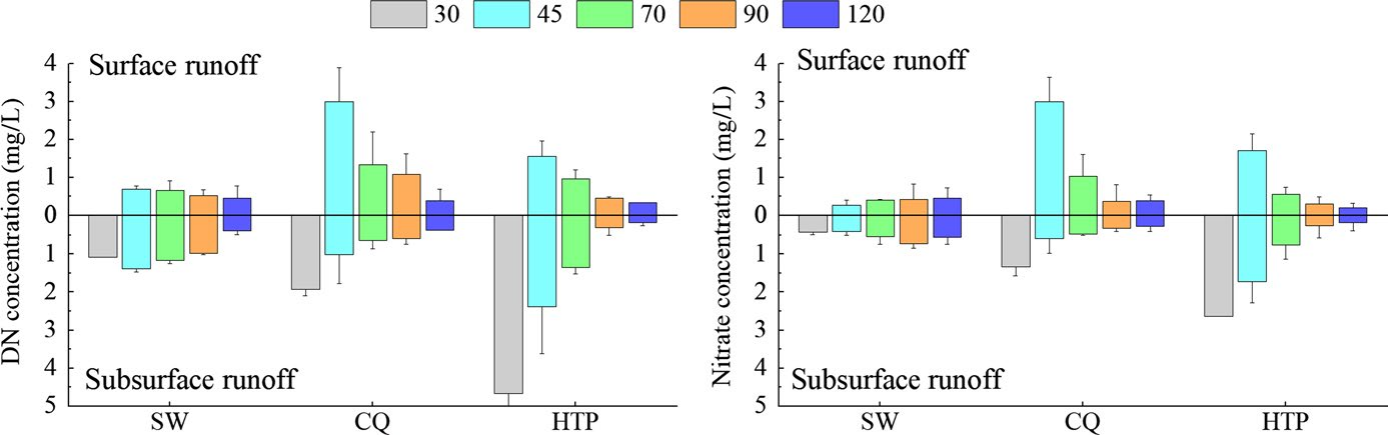
DN and nitrate concentration in surface runoff and subsurface runoff at three sites under different rainfall intensities

Analysis of the nitrogen content in the surface runoff and subsurface runoff showed that the soil lost N mainly in the form of NO_3_^‐^‐N, and accounts for 60%–95% of DN concentration. Concentrations of NH_4_^+^‐N in the soil were generally lower than 1 mg/L (for both surface and subsurface runoff) (Figure 7). The mean [NH_4_^+^‐N] in surface runoff was 0.12, 0.33, and 0.17 mg/L for SW, CQ and HTP, respectively (square brackets denote concentration). In comparison, the mean [NH_4_^+^‐N] in subsurface runoff was 0.21, 0.35, and 0.36 mg/L for SW, CQ and HTP, respectively.

Dissolved nitrogen concentration in surface runoff and subsurface runoffs (Figure 7) at CQ and HTP tended to decrease as simulated rainfall intensity increased. At SW, [DN] in surface runoff (0.46 mg/L as a mean value) and subsurface runoff (1.0 mg/L as a mean value) remained a low level despite increasing rainfall intensity. The [DN] in the surface runoff was higher than that found in subsurface runoff at CQ, whilst SW and HTP showed the inverse correlation (*p *<* *0.05). Nitrate shows a similar change trend with DN as nitrate is main constituent of DN. Both DN and nitrate indicate that N‐loss through subsurface runoff take an important role on N‐loss from karst soil.

#### 3.3.2 Correlation between rainfall and [DN]

**Figure 8:**
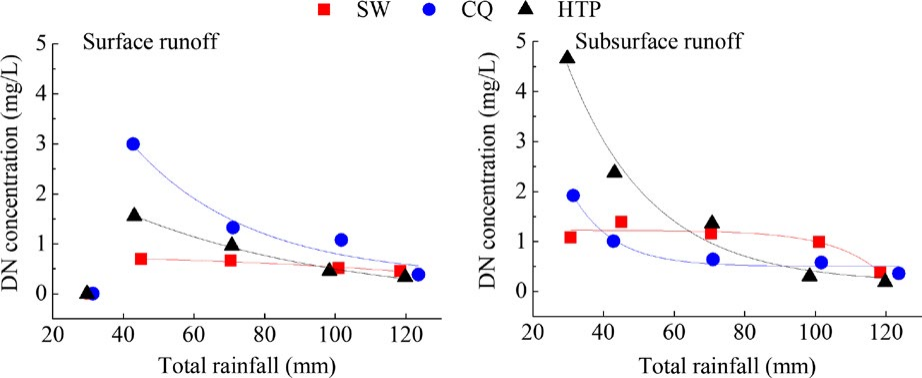
Exponential fitting of [DN] with the amount of simulated rainfall

To find out the correlation between rainfall and [DN], we train three fitting models according formula (5). First‐order exponential decay best describes the correlation between [DN] and rainfall (mm) (Figure 8), which is likely to be associated with the dilution affect (Huebsch et al., 2014). The equations best describing surface and subsurface runoffs at CQ are as follows:

(10) $$C_{1}=9.49\times \exp\left(-P/33.53\right)+0.32\left(r{}^{2}=0.89\right)$$

(11) $$C_{2}=20.47\times \exp\left(-P/11.76\right)+0.50\left(r^{2}=0.95\right)$$

where Equation (10) and (11) belong to surface runoff and subsurface runoff, respectively. The N‐loss at SW did not vary considerably under different rainfall intensities. This may have been a result of a coarser soil (larger particles) and greater slope on this site, compared to the two other sites (Table 2).

### 3.4 N‐loss load at experimental sites

The [DN] measurements were used to calculate the N‐loss at our experimental sites (Table 4). N‐loss load tended to increase with rainfall intensity, then decrease. The highest N‐loss was found when rainfall was 120 mm/hr at all three sites. The highest N‐loss at SW and CQ was from subsurface runoff; mean loss was 3–4 times higher than the surface load. At HTP, the N‐loss load was higher for surface runoff than subsurface runoff significantly (*p *<* *0.05).

**Table 4:** N‐loss load at experimental sites (mg/m^2^)

Rainfall intensity (mm/hr)	SW		CQ		HTP	
	Surface	Subsurface	Surface	Subsurface	Surface	Subsurface
30	–	46.02	–	18.35	–	19.26
45	11.28	56.44	6.73	65.85	4.46	30.61
70	40.05	50.82	12.41	65.76	47.13	17.72
90	36.17	52.31	13.29	75.62	72.72	15.34
120	36.50	55.04	12.11	81.56	77.26	14.85
Average value	24.80	52.13	8.91	61.43	40.31	19.56

### 3.5 Estimate of the N‐loss from karst soil in China

The *P*_min_ calculated by Equation (6) is 3.0 mm, which means the minimum rainfall depth that will trigger runoff at all sites under different rainfall intensities. Due to rapid movement of water in KCNs along with N leakage, N‐loss may have already occurred from top to bottom soil layer inwardly. Different with *P*_c_ which defined as a critical threshold that will trigger runoff at in situ plots, *P*_min_ implied the potential threat and a critical threshold that will trigger N‐loss. In consideration of that it is extremely difficult to acquire detailed data around the whole country, we use *P*_min_ as a regional critical parameter to get rid of daily precipitation data partly and *P*_month_ is a summation of remaining daily data as Equation (7) shows. We calibrate *P*_min_ of North China and Plateau karst area to be 3.5 mm, which at South China is 3.0 mm, according to different soil moisture and precipitation (Zhou et al., 2005).

**Figure 9:**
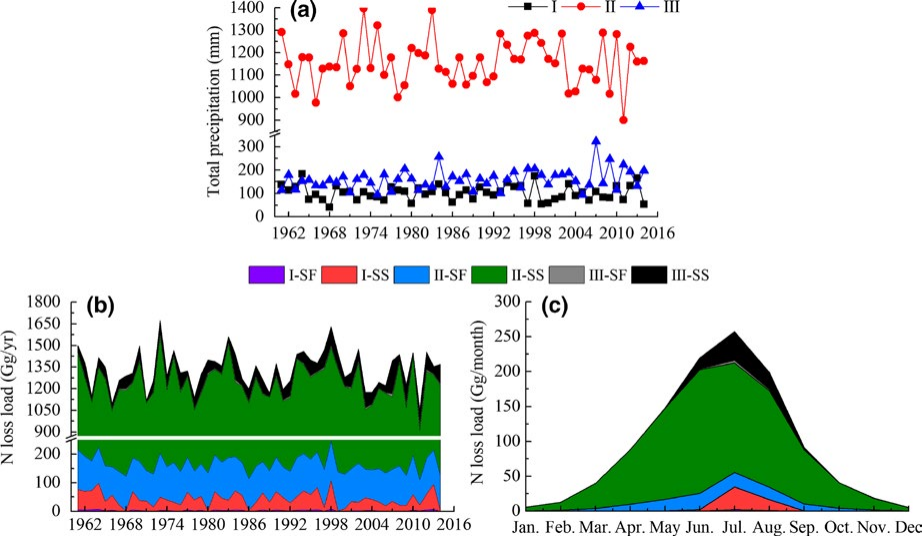
(a) Average annual precipitation of three karst regions in China; (b) Interannual variation of accumulated N‐loss load by regions; (c) Monthly variation of accumulated N‐loss load by regions. SF means surface runoff and SS means subsurface runoff. Roman numerals I, II, III represent three karst areas

Figure 9a shows the mean precipitation for each of the three karst regions after calibration. Region II received the most precipitation (1,158 ± 104 mm/year), followed by Region III (160 ± 43 mm/year) and Region I (102 ± 32 mm/year). These values were used to estimate the N‐loss from the three karst regions. However, for that estimated to be calculated the following assumptions are made as follows:

1. Three karst regions are divided according to soil water content, precipitation, and carbonate rock distribution in China (Jiang et al., 2011; Zhou et al., 2005);
2. The three karst regions can be further divided into dolomite and limestone, in proportion to the overall distribution;
3. Corrected monthly precipitation data were employed to run the estimated model of three regions.

SW data were used to predict runoff in dolomite areas, and CQ data to predict runoff in limestone areas. Correlations between rainfall and runoff and [DN] were used to estimate N‐loss in three karst regions of China. The model was applied to each year between 1961 and 2014 and assumes there has been no major change in land use during the period of 1961–2014. Figure 9b,c summarize the interannual and monthly variation of N‐loss by region disaggregated and by pathway (SS or SF). Region II showed the highest N‐loss (II > III > I, *p *<* *.01). This was also true of both surface and subsurface runoffs and N‐losses. The mean N‐loss was 43 ± 25, 942 ± 99, and 80 ± 38 kg km^−2^ year^−1^ for region I, II and III, respectively.

Region II had the highest rainfall and also the highest N‐loss. Note the high variation, due to rainfall variance over the course of a year. Both N‐loss load and rainfall were highest in the wet season (April to September). In region II, the highest rainfall and N‐loss load were seen in June. In regions I and III, this maximum was seen in July (Figure 9c).

According to our model, N‐loss load in the wet season would have accounted for 89% of the annual load. Yue et al. (2015) studied the similar karstic agricultural field in the Houzhai catchment, Guizhou Province, Southwest China recently. That research state that approximately 85% of nitrate transport occurs during the wet season by isotope analysis of ^15^N and ^18^O in NO_3_^−^, which result is in great accordance with our research (Yue et al., 2015). As a whole, the annual N‐loss is fluctuated at a range of 1.05–1.67 Tg·N year^−1^ during 1951 to 2014. The N‐loss in South China is significantly higher than north and plateau area (*p *<* *.01), with region II accounting for 90% of the total for the China's karst region.

## 4 DISCUSSION

### 4.1 Rainfall threshold of KCN

A recent study at a karst spring in Opacac, Croatia discovered a rainfall‐runoff model, which could be described by the following formula (Zeljkovic and Kadic, 2015):

(12) $$P=Q_{\mathrm{SF}}+Q_{\mathrm{SS}}+R_{G}+R_{S}+R_{E}+\mathrm{ET}$$

where *R*_G_ is underground water recharge through deep percolating (mm); *R*_S_ is soil and rock water storages (mm); *R*_E_ is the water absorption and storage by epikarst; ET is evapotranspiration from the karst surface (mm). In long‐term observational experiments, *R*_E_ and ET are important pathways (Jukić and Denić‐Jukić, 2009); however, they have minimal influence over the course of a 60 min experiment. Therefore, we do not consider ET further in this study. Furthermore, runoff coefficients differ under different rainfall intensities, which means that rainfall can turn into *R*_S_ and *R*_G_ except for *Q*_SF_ and *Q*_SS_. In theory, we think there is a rainfall threshold for producing surface runoff at karst ecosystem, and any rainfall that exceeds the threshold will lead to surface runoff yielding (Semttem et al., 1991). We infer that *R*_S_ and *R*_G_ may be responsible for the threshold. A linear fit to our results confirms that each site showed a critical threshold at which subsurface runoff stops increasing. Therefore, according to the fitting results in Figure 6, the critical threshold (*P*c) are 28.59, 24.88, and 35.89 mm for SW, CQ, and HTP, respectively.

Rainfall mainly transforms into *Q*_SS_, *R*_S_ and *R*_G_ when rainfall is below *P*c. *Q*_SF_ could be as low as zero if rainfall is extremely low. When rainfall is higher than *P*c, rainfall could transforms into *Q*_SF_ *, Q*_SS_ *, R*_S_ and *R*_G_. A study of Mediterranean karst cave below a 143 m^2^ plot showed unsaturated zone includes soil and epikarst or subcutaneous zone may be seen as important buffer controlling the onset and magnitude of water percolation and recharge (Lange et al., 2010). *P*_c_ is an important parameter in the rainfall‐runoff conversion on water and soil loss research.

### 4.2 Appreciable N‐loss of karst area in China

N‐loss from surface and subsurface runoff, if estimated based on our simulated rainfall experiments, would approximate 10% surface and 90% subsurface during this period. This estimate is supported by previous studies to some extent (Buda and DeWalle, 2009; McCormack et al., 2014) . Buda and DeWalle (2009) studied NO_3_^−^ runoff pathway using two‐component mixing models based on stable isotopes of NO_3_^−^ (δN^15^‐NO_3_^−^ and δO^18^‐NO_3_^−^) and water (δO^18^‐H_2_O) from baseflow to peakflow. Results suggested that ground water flow pathways likely flushed stored NO_3_^−^ sources into the stream and soils have promoted NO_3_^−^ assimilation. N‐loss in surface runoff is consistently lower than that from subsurface runoff in all regions mainly due to low surface runoff coefficient. In northwestern Guangxi (located in South China), the surface runoff coefficient was found to be lower than 5%, based on results from a much larger experimental site (>1,000 m^2^) (Chen et al., 2012). This implied that N‐loss mainly moves along with subsurface runoff in South China karst area.

N limitation to primary productivity has different effects on different types of plant communities in karst ecosystems in Southwest China. Zhang et al. (2015) found that the grassland is N limited, the shrubland is constrained by N and P together or other nutrients, which infer that N limitation is one of the key factors during a secondary succession following farmland abandonment. Kang et al. (2015) confirmed the N limitation for DNA synthesis and cellular functions in a group of plants at karst area.

On the other hand, widespread use of N‐based chemical fertilizers (averaging 113–1,124 kg Nha^−1^) may greatly aggravate the dangerous of agricultural nonpoint N pollution (Liang et al., 2005). As karst aquifers are particularly vulnerable to contamination, the fertilizers will be a potential source of pollution especially at unconfined karst zone (Różkowski and Różkowski, 2016).

### 4.3 Challenge of N‐loss estimation at karst area

We collected subsurface runoff at the interface between rock and soil, which is about 60 mm depth. Therefore, the subsurface runoff we collected derived from rain filtrated by the covered soil, and the recharge water which was controlled by deep percolation through fissures or channels was unable to collect. As a result, the N‐loss load estimated this research includes most DN loss from upper soil, not counting DN loss accompanied by deep percolation.

We used monthly mean rainfall data instead of individual rainfall data in the estimated model because we consider the amount rainfall to be main controller of N‐loss. More experiments at north China and Plateau karst area would allow us to improve the accuracy of parameters and refine our models. Moreover, we carried out the simulated rainfall experiment at wet season that the N deposition and soil water content may be differ with that during dry season. So, the estimated results of N‐loss maybe bigger than that practical loss, which should be confirm by another series of experiments under different soil water contents.

Another component for thorough assessment would be the use of radionuclides to determine the range of soil loss and therefore soil capacity (e.g., track soil redistribution under various rainfall conditions by labeling method with ^35^S). It is possible that the redistribution of soil caused by stronger rainfall may migrate fractional nitrate and ammonia nitrogen. It is also possible that frequent or continuous rainfall during the wet season also contributes to lower N‐loss concentration during storms (e.g., lower capacity for episodic events). And it is hard challenge to determine the amount of fractional nitrate and ammonia nitrogen loss because of sedimentation at a larger scale (Gao et al., 2016).

### 4.4 Policy implications

A pair of problems caused by N‐loss are N limitation and N contamination due to the rapid movement of water in KCN. And the fertilizers used for agriculture and ecological restoration would bring more serious nonpoint N pollution as a vicious feedback. The critical question is that N‐loss along with rapid movement of water. Overall control of N‐loss during rainfall‐runoff process maybe an effective strategy. Firstly, using organic fertilizer and slow‐release chemical fertilizer instead of fast‐release chemical fertilizer, avoiding excess fertilization could reduce N‐loss from the source. Secondly, improving soil structure and increasing vegetation coverage could enhance water conservation ability. Nitrogen‐fixing plants may be helpful on ecological restoration of karst ecosystem. Lastly, rainfall and surface runoff stored by small water storage project could be used for irrigation and preventing excessive N‐loss.

## 5 CONCLUSIONS

Rainfall‐runoff conversion process at karst area takes a vital role on sustainable development of healthy ecosystem function. Soil water conservation capacity is particularly important as rainfall is dominating source of water at karst system. Building rainwater storage and water‐saving projects according to characteristics of runoff at different rock types under the shallow soil will contribute to ecological restoration and improve soil productivity.

N‐loss along with runoff from karst soils to water body is significantly higher in karst regions than in non‐karst areas. We estimate that the annual N‐loss ranged from 1.05 to 1.67 Tg/year between 1961 and 2014. South China karst area suffers from more threat of N‐loss than other karst regions, and it is worthy of drawing national attention.

## CONFLICT OF INTEREST

None declared.

## AUTHOR CONTRIBUTION

Song XW and Gao Y proposed the initial idea. Green, Sophie M and Dungait, Jennifer A J designed the study. Peng Tao and Quine, Timothy A conducted the data analyses. Xiong, Bailian, Wen XF and He NP performed the model simulation.
